# The role of MMPs in intracerebral hemorrhage

**DOI:** 10.3389/fcell.2025.1667228

**Published:** 2025-12-17

**Authors:** Xiaofei Guan, Meiren Li, Huimin Li, Zeqin Guo, Muhammad Saad Ullah, Xiaoqun Liu, Moxin Wu, Wenmin Yu

**Affiliations:** 1 School of Health Science and Engineering, University of Shanghai for Science and Technology, Shanghai, China; 2 The School of Basic Medical Science, Jiujiang University, Jiujiang, Jiangxi, China; 3 Affiliated Hospital of Jiujiang University, Jiujiang, Jiangxi, China; 4 Faculty of International Studies, Jiujiang University, Jiujiang, Jiangxi, China; 5 Department of Respiratory and Critical Care, School of Clinical Medicine, Jiujiang University, Jiujiang, Jiangxi, China; 6 Department of Medical Laboratory, Affiliated Hospital of Jiujiang University, Jiujiang, Jiangxi, China; 7 Jiangxi Provincial Key Laboratory of Cell Precision Therapy, Jiujiang University, Jiujiang, Jiangxi, China

**Keywords:** intracerebral hemorrhage (ICH), matrix metalloproteinases (MMPs), blood-brain barrier (BBB), cerebral edema, neuroinflammation

## Abstract

Intracerebral hemorrhage (ICH) is characterized by the disruption of cerebral vascular integrity, leading to hematoma enlargement, edema formation and physical damage to brain tissue. It has an extremely high disability rate and mortality rate, with mortality rates as high as 50%, imposing considerable physical and economic burdens on patients and their families. Therefore, identifying effective therapeutic targets for ICH has become an urgent issue. In recent years, numerous animal and clinical studies have shown that matrix metalloproteinases (MMPs), particularly MMP-9 and MMP-2, are closely associated with the pathophysiological processes of ICH. During the acute phase of ICH, MMP expression increases, leading to the disruption of the blood-brain barrier (BBB), exacerbating neuroinflammation and cerebral edema. However, in the subacute and chronic phases, MMPs play a crucial role in BBB repair, angiogenesis, and neurological recovery. Therefore, MMPs hold promise as effective therapeutic targets for ICH. This article provides an overview of ICH, the primary structure, classification, regulation, and role of MMPs in the destruction of the BBB, angiogenesis, and neural repair in ICH.

## 1 Overview of ICH and mechanism of neuroinflammation

**FIGURE 1:**
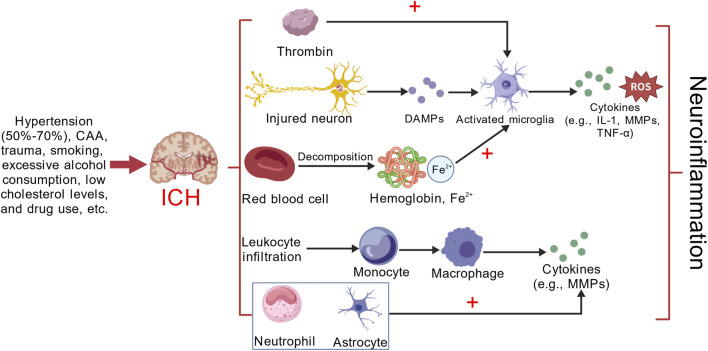
Microglial activation following ICH primarily occurs through three main mechanisms: (1) Hemoglobin, catalyzed by heme oxygenase, degrades into heme and iron, promoting microglial activation and neuronal death. This process also leads to the release of various DAMPs. (2) Thrombin directly activates microglia. (3) Leukocyte infiltration, with monocytes entering the CNS and differentiating into macrophages within the brain parenchyma. Microglia, macrophages, neutrophils, and astrocytes subsequently release a cascade of cytokines, exacerbating neuroinflammation. This figure provides a simplified representation, focusing only on some of the inflammatory cells and cytokines involved in neuroinflammation after ICH. Created with BioGDP.com (Jiang et al., 2025).

ICH is a type of stroke characterized by bleeding due to the rupture of blood vessels within the brain tissue. This condition represents about 10%–20% of all stroke cases and is frequently associated with significant disability or death. ICH is particularly prevalent among specific demographics, including older adults, men, and individuals from low- and middle-income countries, as well as among Asian populations (Xue and Yong, 2020). ICH is associated with a high mortality rate, exhibiting a 40% mortality rate within the first month and a 54% rate within the first year. Among survivors, only 12%–39% achieve long-term functional independence. Key risk factors for ICH include hypertension, cerebral amyloid angiopathy (CAA), trauma, smoking, excessive alcohol consumption, hypocholesterolemia, and substance abuse. Notably, patients with hypertensive ICH account for 50%–70% of all ICH instances. The symptoms of ICH typically progress rapidly, often within minutes to hours, and are predominantly located in the basal ganglia. Common manifestations include headache, nausea, and vomiting. Headaches are particularly common in patients with larger hematomas, whereas smaller deep hematomas rarely induce headache symptoms. Vomiting is especially prevalent in cases of cerebellar hemorrhage, occurring in approximately 50% of ICH patients, and around 10% may experience epileptic seizures (An et al., 2017). Primary brain injury in ICH predominantly occurs due to mechanical compression and increased intracranial pressure from bleeding. As blood components, such as red blood cells and plasma, seep into brain tissue from ruptured vessels, a hematoma forms, exerting pressure on surrounding brain structures. This compression leads to localized ischemia and functional deficits. The mechanical damage disrupts the integrity of brain tissue, resulting in neurological impairments that may also trigger herniation. In the aftermath of ICH, secondary brain injury mainly encompasses neuroinflammation, oxidative stress, BBB disruption, and cellular toxicity, all of which are mediated by various cytokines (Zhubi et al., 2025). MMPs play a significant role in neuroinflammation and BBB disruption (Fan et al., 2025). The following discussion centers on the neuroinflammatory processes that occur after ICH. Neuroinflammation encompasses a wide array of inflammatory cells, such as microglia, neutrophils, and macrophages, along with diverse cytokines, including tumor necrosis factor-α (TNF-α), interleukin-1β (IL-1β), interleukin-6 (IL-6), interleukin-10 (IL-10), and MMPs (Ohashi et al., 2023) (Figure 1). Upon entering brain tissue, red blood cells rupture and release hemoglobin. This hemoglobin is subsequently broken down into heme and iron under the catalytic action of heme oxidase, both of which are cytotoxic. The released heme and iron activate microglia via the TLR4/NF-κB signaling pathway, leading to an increase in the production of proinflammatory factors. In experimental studies of intracerebral hemorrhage (ICH), inhibiting TLR4 has been shown to mitigate neuronal loss, decrease edema formation, and enhance neurological function (Mracsko and Veltkamp, 2014). Neuronal death triggers the release of several damage-associated molecular patterns (DAMPs), including ATP, uridine, and heat shock proteins (HSPs), which in turn activate microglia. Furthermore, microglial activation can also occur through proinflammatory cytokines, chemokines, and additional signaling pathways (Ju and Hang, 2025). Activated microglia secrete a variety of substances, including reactive oxygen species (ROS), reactive nitrogen species (RNS), cytokines, chemokines, and MMPs. These compounds compromise the integrity of the BBB, facilitate the recruitment of monocytes into the central nervous system (CNS), and promote their differentiation into macrophages within the brain tissue. This process intensifies neuroinflammation (Bai et al., 2020). Thrombin plays a pivotal role in the blood clotting mechanism and can be swiftly activated following an ICH. Its activation leads to direct disruption of the BBB via the protease-activated receptor-1 (PAR-1) signaling pathway, resulting in cerebral edema and neuronal injury. Additionally, thrombin has the capacity to activate microglia, which in turn triggers the release of proinflammatory cytokines (Guan et al., 2004; Liu J. C. et al., 2021). The pathological process following ICH is dynamic and multi-stage, typically divided into an acute phase dominated by destruction and a recovery phase dominated by repair. The acute phase generally encompasses the first week following a hemorrhage, with a particular focus on days 1–3. During this critical period, several significant processes occur, including the expansion of the hematoma, disruption of the blood-brain barrier, formation of intracerebral edema, and the onset of neuroinflammation. These events collectively represent a peak period for secondary brain injury (Harkins et al., 2022). The subacute phase usually occurs between the seventh day and the first month after bleeding. During this time, the body starts to absorb the hematoma, the inflammatory response begins to subside, and tissue repair processes are activated. In contrast, the chronic phase typically lasts beyond 1 month after the bleeding event and can continue for several months or even years (Zhang et al., 2025). During the acute phase of ICH, MMPs play a dual role. Initially, they degrade and remodel the extracellular matrix (ECM), disrupt the BBB, initiate inflammatory responses, and contribute to brain tissue damage. However, as the condition progresses into the subacute and chronic phases, MMPs shift their role to support recovery. They promote angiogenesis, facilitate BBB repair, encourage myelin regeneration, and aid in axonal regeneration, all of which are essential for neurological recovery (Rosenberg, 2002; Xue and Yong, 2008; Jiang et al., 2025).

## 2 Matrix metalloproteinases

### 2.1 The structure and classification of MMPs

MMPs or matrix metalloproteinases, are zinc-dependent enzymes that are part of the Metzincin protease family. These enzymes are predominantly secreted by a diverse array of cell types, including fibroblasts, osteoblasts, endothelial cells, vascular smooth muscle cells, macrophages, neutrophils, lymphocytes, syncytial trophoblasts, as well as various cells associated with connective tissue and inflammatory responses (Bassiouni et al., 2021). They are capable of degrading collagen substrates and non-collagenous ECM substrates (Table 1), and also play a crucial role in maintaining vascular integrity, primarily distributed throughout most connective tissues (Cabral-Pacheco et al., 2020). According to the order in which the genes were discovered, they were named MMP-1, MMP-2, MMP-3 ……MMP-28, respectively (Löffek et al., 2011). Currently, 24 types of MMPs have been identified in the human body (Rempe et al., 2016). Based on substrate specificity and the structural organization of their domains, MMPs can be classified into six categories: (1) collagenases (MMP-1, MMP-8, MMP-13, MMP-18) primarily degrade native collagen fibers, participate in tissue remodeling and repair, and maintain extracellular matrix homeostasis through specific collagen degradation (Ulrich et al., 2010). (2) Gelatinases (MMP-2, MMP-9) primarily contribute to cell migration, angiogenesis, and tumor invasion (Venkataraman et al., 2016). They are often secreted as inactive proenzymes and, upon activation, degrade denatured collagen and gelatin (de Almeida et al., 2022). (3) Stromelysins (MMP-3, MMP-10, MMP-11) promote cell migration, tissue remodeling, and inflammatory responses by degrading multiple ECM components (Yamamoto, 2013; Reddi et al., 2025). Additionally, MMP-3 hydrolyzes α1-antitrypsin. (4) Matrilysins (MMP-7, MMP-26) exhibit broad substrate specificity. These enzymes are primarily involved in tissue repair, inflammatory responses, and tumor metastasis (Khamis et al., 2013; Tuomainen et al., 2014). (5) Membrane-type MMPs (MT1-6-MMPs, i.e., specifically MMP-14, -15, -16, -17, -24, and -25, are enzymes anchored to cell membranes. This group of enzymes plays a crucial role in activating MMP-9 and MMP-13, thereby facilitating cell migration and invasion through the targeted degradation of the extracellular matrix. Additionally, other matrix metalloproteinases, including MMP-12, -19, -20, -21, -23, -27, and -28, primarily contribute to tissue remodeling, inflammatory processes, and tumor metastasis (Table 1) (Cui et al., 2017). MMPs possess a well-conserved structural architecture characterized by five functional domains that extend from the N-terminus to the C-terminus: a hydrophobic signal peptide, a pro-domain, a catalytic domain, a proline-rich hinge region, and a C-terminal domain. Among these, the catalytic and pro-domains show remarkable conservation across the MMP family. The hydrophobic signal peptide, a brief amino acid sequence, plays a crucial role in facilitating the entry of MMPs into the cellular secretory pathway. The pro-domain is responsible for keeping the enzyme in an inactive zymogen state; it includes a cysteine residue that coordinates with a zinc ion in the catalytic site, thus inhibiting enzymatic activity. Notably, this domain features a conserved “cysteine switch” motif, where the cysteine residue interacts with the zinc ion in the catalytic domain to maintain the enzyme’s latent, inactive conformation (Wang and Khalil, 2018). The catalytic active site is the essential element of MMPs, playing a pivotal role in the hydrolysis of protein substrates. At its core lies a zinc-containing active center, with zinc ions being crucial for the catalytic function of MMPs. This catalytic domain comprises approximately 160–170 amino acid residues and demonstrates a high degree of sequence similarity across different MMPs. Connecting the catalytic active site to the carboxy-terminal region is the hinge region, which provides the necessary flexibility for MMPs to interact effectively with their substrates. The carboxy-terminal region is linked to substrate specificity and may also be involved in interactions between the enzyme and cell surface receptors. Notably, MMP-2 and MMP-9 feature three fibronectin type II-like motifs in this region, which serve as tightly packed collagen-binding domains, exhibiting a strong affinity for collagen α1 chains (de Almeida et al., 2022; Alexander et al., 2024).

**TABLE 1:** Classification, functions, and characteristics of MMPs.

Classification	Representative members	Substrates	Functions	Characteristics
Collagenases	MMP-1 MMP-8 MMP-13 MMP-18	Collagen types I, II, III, IV, V, VII, VIII, X, XI, gelatin, aggrecan, fibronectin, laminin, tenascin, versican	Degrade native fibrillar collagens, participate in tissue remodeling and repair	Specific for collagen degradation, crucial for maintaining ECM balance
Gelatinases	MMP-2 MMP-9	Gelatin, collagens I, IV, V, VII, X, XI, fibronectin, elastin, laminin, vitronectin, proMMPs-9 and 13	Involved in cell migration, angiogenesis, tumor invasion	Typically secreted as inactive proenzymes, activated to degrade denatured collagen and gelatin
Stromelysins	MMP-3 MMP-10 MMP-11	Proteoglycans, laminin, gelatin, fibronectin, elastin, collagens III, IV, V, IX, X, XI, vitronectin, α1-proteinase inhibitor	Promote cell migration, tissue remodeling, and inflammatory responses	Degrade a variety of ECM components, important for ECM remodeling and cell migration
Matrilysins	MMP-7 MMP-26	Proteoglycans, laminin, fibronectin, gelatin, collagen IV, elastin, tenascin	Involved in tissue repair, inflammatory responses, and tumor metastasis	Secreted enzymes with broad substrate specificity
Membrane-type MMPs	MMP-14 MMP-15 MMP-16 MMP-17 MMP-24 MMP-25	Collagen types I, II, III, gelatin, fibronectin, laminin, proteoglycans, proMMP-2 and 13	Involved in local degradation of the ECM and cell migration	Membrane-bound enzymes that can directly degrade ECM components, facilitating cell invasion and migration
Other MMPs	MMP-12 MMP-19 MMP-20 MMP-21 MMP-23 MMP-27 MMP-28	Gelatin, elastin, fibronectin, laminin, collagen types I, IV, V, aggrecan, tenascin, vitronectin	Involved in tissue remodeling, inflammatory responses, and tumor metastasis	Diverse substrate specificities and functions, often involved in specialized processes

### 2.2 Regulation of MMPs

#### 2.2.1 Activation of MMPs

**FIGURE 2:**
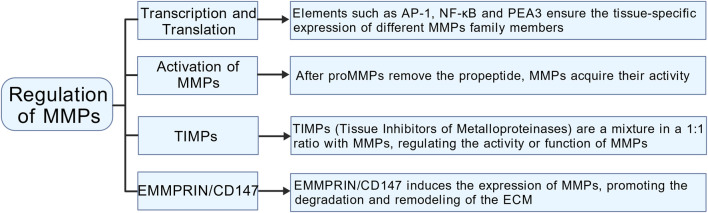
The regulation of MMPs in the human body.

MMPs are synthesized and released as inactive pro-enzymes, known as proMMPs. The activation of MMPs is a complex process that involves multiple steps and mechanisms (Figure 2). Initially, MMPs are translated as pre-proMMPs, and during this translation, the signal peptide is cleaved to produce proMMPs. The critical step in MMPs activation involves the removal of the propeptide, a process typically facilitated by other proteases, such as serine proteases and metalloproteases, or through an autolytic mechanism. Once the propeptide is removed, the catalytic domain undergoes a conformational change, allowing MMPs to become active (Hey and Linder, 2024). Certain MMPs (such as MMP-2 and MMP-9) can be activated by the activation of other MMPs. For example, the activation of MMP-2 typically depends on the action of MMP-14 (Sanyal et al., 2021). Some MMPs possess the capability of autoactivation. For instance, MMP-3 undergoes autocatalytic cleavage, which removes its pro-domain and converts it into an active form. This activation mechanism is crucial for regulating MMP activity and its involvement in various physiological and pathological contexts (Bassiouni et al., 2021).

#### 2.2.2 Regulation of MMPs at the transcription and translation levels

MMP expression is meticulously regulated at both the transcriptional and translational levels, resulting in low enzyme levels during normal tissue homeostasis (Figure 2). Many MMP gene promoters feature conserved cis-acting elements that interact with transcription factors such as AP-1, NF-κB, and PEA3. These factors modulate transcription in response to signals from growth factors or cytokines. The dynamic interplay among these regulatory elements, combined with the integration of multiple signaling pathways, facilitates complex interactions among various transcriptional regulators. This sophisticated regulation guarantees tissue-specific expression patterns for different MMPs and maintains stringent control over their transcriptional activity (Cui et al., 2017). MMP gene transcription is regulated by various epigenetic mechanisms, such as DNA methylation and histone acetylation. Additionally, post-transcriptional regulation plays a crucial role in modulating MMP expression through mechanisms that affect mRNA stability and miRNA-mediated pathways. These pathways can inhibit MMP expression either by repressing transcription or promoting the degradation of mRNA. Moreover, MMPs are also significantly regulated at the translational level, as they are primarily secreted as inactive proenzymes. Within the propeptide domain, the “cysteine switch” creates a steric hindrance at the active site, blocking substrate access and ensuring that the enzyme remains in its inactive form (Madzharova et al., 2019).

#### 2.2.3 Regulation of MMPs by tissue inhibitors of metalloproteinases (TIMPs) and extracellular matrix metalloproteinase inducer (EMMPRIN/CD147)

TIMPs, including TIMP-1, TIMP-2, TIMP-3, and TIMP-4, serve as natural inhibitors of MMPs (Figure 2). By forming stoichiometric complexes with MMPs, TIMPs effectively regulate MMP activity, playing a crucial role in preserving the structural integrity of tissues (Cabral-Pacheco et al., 2020). Lieke Jäkel et al. conducted a study examining the expression levels of MMP-9 and TIMP-3 in patients with cerebral arteriovenous malformations who experienced intracerebral hemorrhage (CAA-ICH) compared to those with CAA without hemorrhage. Their findings revealed that patients with CAA exhibiting elevated levels of MMP-9 also showed decreased levels of TIMP-3 in their cerebral blood vessels. This observed imbalance in the expression of these proteins is linked to the occurrence of secondary intracerebral hemorrhage in individuals with CAA (Jäkel et al., 2020). Chen et al. explored the connection between polymorphisms in the MMP-2 and TIMP-2 gene promoters and the risk of spontaneous deep intracerebral hemorrhage (SDICH) in the Taiwanese population. Their research revealed a significant association between MMP-2 and TIMP-2 promoter variants and susceptibility to SDICH, highlighting notable differences related to age and gender (Chen et al., 2015). The balance between MMPs and TIMPs typically determines the extent of ECM protein degradation and tissue remodeling. Kurogi et al. noted that an increase in TIMP-1 during the initial phase of cerebral vasospasm contributes to the repair of the ECM in the subsequent phase, thereby providing a protective effect against cerebral vasospasm (Kurogi et al., 2015). EMMPRIN/CD147, encoded by the BSG gene located on the short arm of human chromosome 19 (p13. 3), spans approximately 12 kb and comprises 10 exons. It is a heavily glycosylated transmembrane protein known for its significant role as an upstream inducer of MMPs (Patrizz et al., 2020; Liu et al., 2022) (Figure 2). Caveolin-1 acts as a negative regulator of EMMPRIN by interacting with its EC2 domain. This interaction induces conformational changes that reduce both the expression and functionality of EMMPRIN on the cell surface, ultimately resulting in decreased MMP activity (Liu et al., 2023). Following ICH, red blood cell rupture releases hemoglobin, whose degradation generates substantial free radicals—the primary source of ROS (Hu et al., 2016). ROS can increase the MMPs activity via direct and indirect mechanisms (Katsu et al., 2010). ROS species such as hypochlorous acid and peroxynitrite, can directly oxidize the “cysteine switch” in the proenzyme precursors of MMPs. This oxidation modifies their conformation and initiates the process of self-activation (Bassiouni et al., 2021). ROS serve as powerful second messengers that trigger various essential intracellular signaling pathways, including NF-κB and MAPK. The activation of these pathways leads to a substantial increase in the transcriptional expression of genes responsible for encoding matrix metalloproteinases, specifically MMP-2 and MMP-9 (Deng et al., 2024; He et al., 2025).

## 3 The role of MMPs in ICH

### 3.1 Changes in the expression of MMPs in tissues after ICH

#### 3.1.1 MMP-9

Following ICH, microglia, macrophages, neutrophils, astrocytes, and endothelial cells are the primary sources of MMPs. Numerous experimental studies have demonstrated increased levels of MMPs in brain tissue after ICH (Table 2). Among these, MMP-9 has been the most extensively researched, predominantly produced by microglia, macrophages, neutrophils, astrocytes, and endothelial cells. Research conducted by Power et al., revealed a significant rise in MMP-9 mRNA expression at both 24 h and 7 days post-ICH(Power et al., 2003; Tejima et al., 2007). Numerous clinical studies have established that MMP-9 is crucial in the disruption of the BBB. Its expression is notably increased in the tissue surrounding the hematoma (Ke et al., 2007; Wu et al., 2008; Li N. et al., 2013). MMP-9 enhances vascular permeability by breaking down tight junction proteins, including claudin-5 and ZO-1, as well as components of the basement membrane. This degradation contributes to the onset of cerebral edema and neuroinflammation (Abilleira et al., 2003; Rosell et al., 2006; Dang et al., 2017; Noroozi-Aghideh et al., 2019). MMP-9 induced BBB disruption is a major driver of vasogenic edema and neuronal apoptosis (Li Z. et al., 2020). Li et al. discovered that the expression of MMP-9 was notably elevated in patients with ruptured cerebral arteriovenous malformation (AVM) when compared to those with unruptured AVMs and the control group. This finding suggests a potential link between MMP-9 levels and secondary injury that may occur following ICH (Li X. et al., 2013). In a study by Ji et al., L13 protected the integrity of basement membranes and tight junction proteins by inhibiting MMP-9 activity, further confirming its critical role in the acute pathological process of ICH (Ji et al., 2023). Moreover, miR-195-5p notably reduced neurological damage, cerebral edema, and BBB disruption following ICH by suppressing the expression of MMP-9. This suggests that MMP-9 could serve as a promising target for therapeutic intervention (Tsai et al., 2024).

**TABLE 2:** Expression changes of MMPs in patients with ICH.

Study	Study popultion	Main findings
Li X. et al. (2013)	31 patients	The expression level of MMP-2 in the ruptured group (arteriovenous malformation, AVM) was significantly higher than that in the unruptured group and the control group, the levels of IL-6 in the blood correlated with the tissue levels of activated MMP-9
Xia et al. (2021)	68 patients	The serum levels of MMP-2 in CAA-ICH patients were significantly decreased, while the levels of MMP-9 were significantly increased. There was no significant difference in MMP-3 levels between the experimental group and the control group
Yu et al. (2023)	93 patients	The plasma MMP-2 level of ICH patients was significantly lower than that of the healthy control group
Ke et al. (2007)	42 patients	The positive expression of MMP-2 and MMP-9 in the localized brain tissues of patients with early acute cerebral hemorrhage significantly increased
Tsai et al. (2024)	ICH rats plus miR-195-5p	miR-195-5p has significant neuroprotective effects against ICH-induced neuronal injury by downregulating the expression of MMP-9 and MMP-2
Howe et al. (2018)	79 patients	The expressions of MMP-1, MMP-3, MMP-8, MMP-9 and MMP-10 all increased after cerebral hemorrhage
Howe et al. (2019)	55 patients	Among male patients, the levels of MMP-1, MMP-2, MMP-3 and MMP-9 gradually increased over time until 10 days after the injury. However, among female patients, MMP-8 was the only subtype that showed significant changes over time, with its peak occurring 3–5 days after the injury
Li N. et al. (2013)	59 patients	MMP-3 and MMP-9 significantly increased after cerebral hemorrhage. The increase in MMP-3 levels was independently associated with the volume of edema around the hematoma

#### 3.1.2 MMP-2

Unlike MMP-9, MMP-2 exhibits constitutive expression in normal brain tissue, with its activity significantly “induced” following ICH. In studies by Power et al., MMP-2 mRNA expression showed marked elevation on day 7 post-ICH (Power et al., 2003; Tejima et al., 2007). Xia et al. found that patients with CAA-ICH had significantly lower serum levels of MMP-2. Notably, these reduced MMP-2 levels correlated with an increased risk of ICH recurrence. This observation suggests that MMP-2 may play a protective role in cognitive function (Xia et al., 2021). Yu et al. discovered that plasma levels of MMP-2 were significantly lower in patients with ICH compared to healthy controls. This reduction in MMP-2 levels correlated with increased severity of cerebral edema and worse neurological outcomes. These findings further underscore the role of MMP-2 in the inflammatory responses and tissue repair mechanisms that occur after ICH (Yu et al., 2023). Ke et al. found that the expression levels of MMP-2 were significantly higher in focal brain tissue from patients experiencing early acute intracerebral hemorrhage (ICH). This finding suggests that MMP-2 may play a crucial role in the processes of neuroinflammation and the development of cerebral edema (Ke et al., 2007). In the subacute and chronic phases of ICH, the reduction in stimulatory signals (such as hemoglobin and thrombin), the shift of primary MMP-2 secreting cells (e.g., activated microglia/macrophages) from the proinflammatory M1 phenotype to the anti-inflammatory/reparative M2 phenotype (Wu et al., 2025), and the binding of TIMP-2 to MMP-2 leading to its inactivation collectively contribute to decreased MMP-2 expression levels (Dzwonek et al., 2004).

#### 3.1.3 Other MMPs

Ke et al. found that the expression levels of MMP-2 were significantly higher in focal brain tissue from patients experiencing early acute ICH. This finding suggests that MMP-2 may play a crucial role in the processes of neuroinflammation and the development of cerebral edema (Power et al., 2003; Tejima et al., 2007). In the study by Howe et al., MMP-1, MMP-3, MMP-8, and MMP-10 were elevated after ICH. MMP-8 and MMP-1 were significantly correlated with subacute-phase (6–8 days) cerebral edema and delayed neurological deterioration, MMP-3 was associated with acute neurological deterioration (END, within 24 h), and MMP-10 was significantly correlated with hematoma expansion (within 2 days) (Howe et al., 2018). This study highlighted significant gender differences in the expression of MMPs. In male patients, the levels of MMP-1, MMP-2, MMP-3, and MMP-9 increased steadily until day 10 post-injury. Conversely, female patients exhibited notable changes solely in MMP-8, with levels reaching their peak between days 3 and 5 (Howe et al., 2019). Li et al. also observed that MMP-3 levels were independently correlated with the volume of perihematoma edema (Li N. et al., 2013). In the study conducted by Suzuki et al., researchers observed a notable increase in MMP-3 expression in the capillary endothelial cells located in the infarct zone of mice after administering tPA treatment. This finding implies that MMP-3 may facilitate hemorrhagic transformation by degrading the basement membrane of neurovascular units (Suzuki et al., 2007). MMP-19 and MMP-26 expression significantly rose after CAA-associated ICH, especially within vascular amyloid deposits. This observation suggests a possible role for these enzymes in CAA pathology. However, further research is needed to determine whether this increase is directly caused by ICH (Tanskanen et al., 2011).

### 3.2 The role of MMPs in secondary injury following ICH: from BBB disruption to neuroinflammation

**FIGURE 3:**
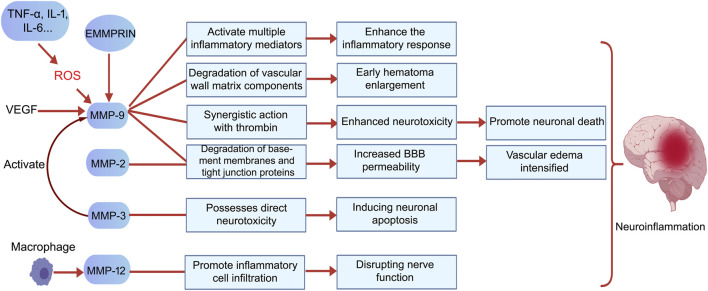
MMPs exert harmful effects following ICH primarily by promoting inflammatory responses, exacerbating vasogenic edema, enhancing neurotoxicity, inducing neuronal death, and intensifying neuroinflammation. Specifically, MMP-9 increases BBB permeability by degrading vascular matrix components and tight junction proteins, thereby promoting acute hematoma expansion. It also synergizes with thrombin to enhance neurotoxicity and facilitate neuronal death. Additionally, VEGF induces high MMP-9 expression, which in turn contributes to hematoma enlargement by promoting abnormal vascular proliferation, creating a vicious cycle. MMP-2 and MMP-9 jointly degrade basement membrane components, leading to increased BBB permeability and exacerbated cerebral edema. MMP-3 exhibits direct neurotoxicity by inducing neuronal apoptosis and activating pro-MMP-9, thereby expanding the proteolytic cascade. MMP-12, secreted by macrophages, promotes inflammatory cell infiltration and brain tissue destruction, closely correlating with poor neurological recovery. The activation and functions of these MMPs collectively drive the onset and progression of neuroinflammation. Created with BioGDP.com (Jiang et al., 2025).

Secondary brain injury following ICH is involves a complex interplay of physiological processes, with perihematoma edema (PHE) and neuroinflammation being central mechanisms contributing to neurological decline. In the acute phase, PHE is characterized primarily by cytotoxic edema. In contrast, the later stages of PHE are largely defined by vasogenic edema, which results from the breakdown of the BBB and an increase in vascular permeability (Wan et al., 2023). The blood-brain barrier (BBB) is formed by microvascular endothelial cells, astrocytic terminal processes, pericytes, and the basement membrane. This structure plays a crucial role in preserving the stability of the internal environment within the central nervous system (Lu and Wen, 2024). Multiple MMPs are crucial in disrupting the BBB, facilitating the infiltration of inflammatory cells, causing neuronal injury, and exacerbating neuroinflammation. However, the specific roles of these MMPs differ among their various subtypes. (Figure 3) (Jiang et al., 2025).

#### 3.2.1 MMP-9: a core effector in BBB disruption and neuroinflammation

MMP-9 predominantly contributes to detrimental outcomes during the acute phase of cerebral hemorrhage, particularly in the first 3 days after bleeding begins (Chang et al., 2014). In the study by Alvarez-Sabín et al., MMP-9 reached its peak 24 h after intracerebral hemorrhage and remained elevated between 48 h and 7 days. MMP-9 activity peaked at 7 days and showed a sustained positive correlation with edema volume (Alvarez-Sabín et al., 2004). MMP-9 is recognized as the key protease responsible for causing acute disruption of the BBB and the development of vasogenic edema in ICH. In studies utilizing MMP-9 knockout stroke models, researchers noted significantly higher levels of the tight junction protein ZO-1, which correlated with a substantial decrease in BBB permeability and a marked reduction in cerebral edema. These findings directly underscore the essential function of MMP-9 in these pathological processes (Gu et al., 2011). Its mechanisms primarily include: (1) Direct degradation of BBB structural components: MMP-9 efficiently degrades type IV collagen, fibronectin, and laminin—key constituents of the vascular basement membrane and extracellular matrix (ECM)—thereby disrupting the physical barrier of the BBB (Li et al., 2023). (2) Disruption of tight junctions: MMP-9 degrades tight junction proteins such as ZO-1 and claudin-5, weakening connections between endothelial cells. This directly increases vascular permeability and promotes vasogenic cerebral edema (Gasche et al., 2006; Rosenberg and Yang, 2007; Sandoval and Witt, 2008). (3) Promoting Inflammatory Cell Infiltration: Disruption of the BBB removes the physical obstacles that hinder the migration of leukocytes and macrophages. As these inflammatory cells infiltrate the brain tissue, they begin to release various inflammatory mediators, which intensify neuroinflammation and contribute to additional damage to the BBB (Reijerkerk et al., 2006; Zozulya et al., 2007; Rempe et al., 2016). (4) Mediating neurotoxicity: MMP-9 possesses intrinsic neurotoxicity and exhibits synergistic effects with thrombin, intensifying neuroinflammation and neuronal death (Xue et al., 2006). (5)Positive feedback loop with VEGF: VEGF induces high MMP-9 expression, while MMP-9 promotes abnormal vascular proliferation, contributing to hematoma expansion and forming a vicious cycle (Rundhaug, 2005; Vempati et al., 2014). Neuroinflammatory mediators like TNF-α and IL-6 increase the expression of MMP-9. Furthermore, the reactive oxygen species (ROS) produced during this process amplify MMP-9 activity. Together, these factors contribute to the worsening of secondary injury (Wang and Khalil, 2018).

#### 3.2.2 MMP-2: a dual-potential regulatory factor

MMP-2 is significantly involved in the pathological processes associated with ICH, although its role is complex. Castellazzi et al., found a positive correlation between MMP-2 levels and edema volume measured 24–48 h after the occurrence of ICH (Castellazzi et al., 2010). Similar to MMP-9, MMP-2 can degrade major components of the basement membrane (such as type IV collagen), contributing to acute BBB dysfunction and the formation of vasogenic edema (Li et al., 2023). Clinical studies have observed that plasma MMP-2 levels in ICH patients correlate with cerebral edema severity and neurological outcome (Ke et al., 2007; Yu et al., 2023), suggesting its involvement in acute injury processes. MMP-2 stands out as a constitutively expressed enzyme, differing from the inducible MMP-9. This distinctive feature means that MMP-2 is consistently present in physiological conditions. However, its role becomes more complex in pathological states, where it may contribute to acute destructive processes by degrading blood-brain barrier structures and tight junction proteins. This degradation can lead to hemorrhagic transformation and cerebral edema. While Caveolin-1 negatively regulates MMP-2 activity, changes in the microenvironment can prompt a shift in its function toward reparative processes (Gu et al., 2011). This duality may clarify why it demonstrates protective associations in certain studies. However, its direct effects on neuronal survival and inflammatory pathways are less explored than those of MMP-9, indicating a need for further investigation in this area.

#### 3.2.3 Synergistic and specific actions of other MMPs

In addition to MMP-9 and MMP-2, several other members of the MMP family contribute to pathological damage following ICH through various mechanisms. MMP-3, for instance, enhances proteolytic cascades due to its ability to degrade a wide range of ECM components and activate pro-MMP-9 (Gasche et al., 2006; Li et al., 2023), but has also been demonstrated to possess direct neurotoxicity, inducing neuronal death (Xue et al., 2009). Active MMP-3 released during neuronal apoptosis can further activate microglia, leading to the release of pro-inflammatory factors. This process directly contributes to neuroinflammatory signaling (Kim et al., 2007; Woo et al., 2008). MMP-12 expression is markedly increased following ICH and is strongly linked to suboptimal functional recovery. This relationship is largely due to MMP-12’s role in enhancing the recruitment and activation of inflammatory cells (Wells et al., 2005). Membrane-associated MMPs (e.g., MT4-MMP/MMP-17) directly initiate neuroinflammatory pathways by catalyzing the conversion of membrane-bound TNF-α into soluble, active forms (Dou et al., 2017). MMPs, such as MMP-7, play a crucial role in regulating neuronal apoptosis signaling. They achieve this by cleaving death factors, including Fas ligand, which contributes to neurotoxic processes (Powell et al., 1999). These MMPs, along with MMP-9 and MMP-2, form a complex proteolytic network that interacts with signaling pathways such as Caveolin-1/NF-κB (Cao et al., 2023), synergistically exacerbating BBB disruption, neuroinflammation, and neuronal injury.

### 3.3 The roles of MMPs in angiogenesis and neural repair

**FIGURE 4:**
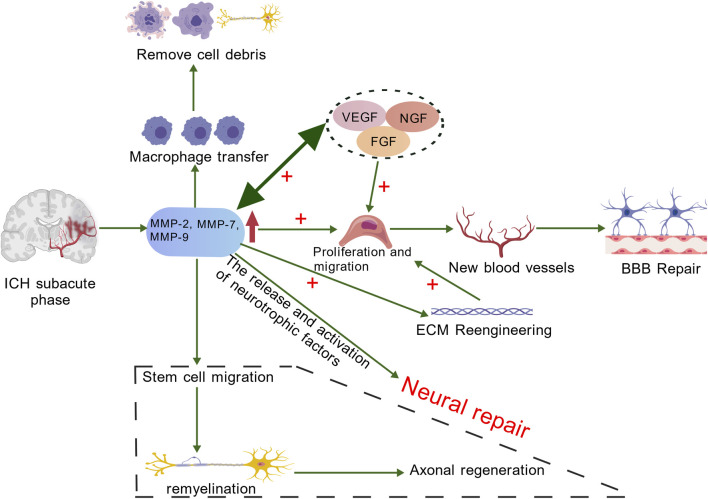
In the chronic stage of ICH, MMPs (mainly MMP-7 and MMP-9) promote the increase of VEGF and FGF, facilitating the proliferation and migration of endothelial cells. MMPs can enhance the activity of these active factors, and angiogenic factors can also induce the expression of MMPs in endothelial cells. The migrating endothelial cells form precursor structures such as bud-like protrusions that promote the repair of the BBB. The remodeling of the ECM not only provides conditions for the migration and proliferation of endothelial cells as well as the synthesis of new matrix components, but also provides space for the regeneration and migration of nerve cells. Neural repair mainly includes endogenous angiogenesis, proliferation and migration of neural stem cells, myelin regeneration and synaptic reconstruction, etc. MMPs play an important role in these processes. Additionally, the activity of MMPs helps macrophages to migrate to the damaged area, thereby promoting the clearance of cell debris during the tissue repair process. Created with BioGDP.com (Jiang et al., 2025).

MMPs are essential for various normal physiological processes, including maintaining tissue homeostasis, facilitating cellular migration, and promoting angiogenesis. Additionally, they are involved in several pathological conditions, such as wound healing, inflammation, and the formation of blood vessels associated with tumors (Quintero-Fabián et al., 2019). During the subacute and chronic phases of cerebral hemorrhage, approximately 7 days post-bleeding and beyond, MMPs degrade damaged extracellular matrix components, with MMP-2 and MMP-9 playing particularly crucial roles in this process (Chang et al., 2014). These enzymes break down collagen and gelatin in the basement membrane, which encourages the movement and growth of newly formed endothelial cells. As the extracellular matrix degrades, resistance in the surrounding environment decreases, allowing for easier migration of neural stem cells and endothelial cells. This process supports the repair of the blood-brain barrier (Page-McCaw et al., 2007). Furthermore, MMPs can activate and release growth factors, supporting angiogenesis and neural regeneration (Rosell and Lo, 2008). Tissue remodeling plays a vital role in recovery ICH. Research indicates that increased levels of growth factors significantly promote angiogenesis, vascular remodeling, and neurogenesis. These processes collectively contribute to effective tissue repair and the restoration of function (Lee et al., 2006). Administering MMP inhibitors within 7 days following a stroke has been found to hinder neurovascular remodeling and worsen ischemic brain injury, underscoring the role of MMPs in neurovascular remodeling during chronic stages of ICH (Zhao et al., 2006). Wang et al. conducted research demonstrating that endothelial cells stimulated by erythropoietin (EPO) promote the migration of neural progenitor cells. This process occurs through the secretion of matrix metalloproteinases MMP-2 and MMP-9, ultimately leading to enhanced neurological recovery following a stroke (Wang et al., 2006). Additionally, MMPs play a crucial role in modulating the actions of both pro-inflammatory and anti-inflammatory cytokines. This regulation is essential for managing the inflammatory environment within the brain and reducing further damage to the BBB (Rempe et al., 2016). During the recovery and healing stages after ICH, MMPs play a crucial role in promoting angiogenesis through two primary pathways. Firstly, MMPs degrade vital components of the vascular basement membrane, including type IV collagen. This degradation is essential for allowing endothelial cells to migrate from existing blood vessels into surrounding tissues. Secondly, MMPs contribute to the remodeling of the ECM, creating an environment that is conducive to endothelial cell migration, proliferation, and the synthesis of new matrix components. In contrast, TIMPs, such as TIMP-2, can impede the proliferation of endothelial cells (Bajbouj et al., 2021). Following an ICH, the ECM undergoes a remodeling cascade in which specific MMPs play a crucial role. Notably, MMP-7 and MMP-9, along with MMP-1, -2, -3, -10, and -11, are involved in the upregulation of VEGF and fibroblast growth factor (FGF) expression. VEGF is pivotal for stimulating the proliferation and migration of endothelial cells, while MMPs enhance the activity of these growth factors. This increase in angiogenic factors leads to further expression of MMPs in endothelial cells, creating a synergistic effect that promotes angiogenesis after ICH (Wang and Khalil, 2018). Ultimately, with VEGF’s assistance, migrating endothelial cells construct preliminary structures of new blood vessels referred to as sprouts, aiding in BBB repair (Rundhaug, 2005). This angiogenic remodeling is necessary but can also be leaky if unregulated, linking back to the dual role of MMPs. Following an ICH, neurological repair primarily involves several key processes: endogenous angiogenesis, the proliferation and migration of neural stem cells, remyelination, and the reconstruction of synapses. MMPs are crucial in facilitating these processes. Additionally, they may promote the release and activation of neurotrophic factors, further supporting the repair of the nervous system (Lattanzi et al., 2020). ECM remodeling is essential for enabling neural cell regeneration and migration. MMPs play a crucial role in this process by breaking down ECM components that hinder axonal growth. This action reduces the barriers to axon regeneration, fostering a more conducive environment for neural repair (Figure 4) (Li et al., 2023). MMP activity also contributes to the recruitment of macrophages to injury sites, promoting the removal of cellular debris during tissue repair. In addition, research has indicated that MMP-9 can aid in remyelination and axonal regeneration during the chronic phases of ICH (Trivedi et al., 2019). In conclusion, MMPs play a crucial role in promoting angiogenesis and facilitating blood-brain barrier repair during the subacute and chronic phases of ICH, thereby enhancing neurological recovery.

### 3.4 Seeking evidence in cerebral ischemia

In cerebral ischemia, MMP-2 and MMP-9 have become research focal points due to their efficient degradation of major basement membrane components (type IV collagen, laminin, and fibronectin) (Rosenberg et al., 1998). Within 3 hours after ischemia, MMP-9 levels rise significantly in both the infarct core and the penumbra, remaining elevated until day seven. The primary sources of this increase are infiltrating neutrophils, activated microglia, and cerebral vascular endothelial cells (Rosell et al., 2006). MMP-9 directly cleaves tight junction proteins such as ZO-1, occludin, and claudin-5. This cleavage results in widened gaps between endothelial cells and heightened vascular permeability, which in turn induces vasogenic cerebral edema and hemorrhagic transformation. Animal studies consistently show that either knocking out MMP-9 or pharmacologically inhibiting its activity significantly reduces blood-brain barrier disruption, decreases infarct volume, and enhances neurological function scores (Asahi et al., 2001b). Some research indicates that MMP-2 plays a significant role in acute BBB injury; however, its absence does not always result in protective outcomes in every model. This inconsistency suggests that MMP-2 may exhibit a spatiotemporal duality in its function (Asahi et al., 2001a). Additionally, MMP-3 and MMP-12 expression is significantly upregulated post-stroke: MMP-3 activates pro-MMP-9, amplifying the proteolytic cascade (Ogata et al., 1992); MMP-12 further exacerbates neurovascular injury by promoting inflammatory cell infiltration (Chelluboina et al., 2015). MMPs primarily have destructive effects during the acute phase, but during the subacute phase, they play a crucial role in vascular remodeling, axonal plasticity, and neural repair (Jayaraj et al., 2019). Delayed administration of broad-spectrum MMP inhibitors unexpectedly worsens neurological deficits, suggesting a biphasic “destruction-repair” functional profile (Yang et al., 2019). This dual role mirrors the contrasting effects of MMPs in ICH.

## 4 MMPs as potential therapeutic targets for ICH

MMPs play a crucial role in secondary injury and recovery processes after ICH, and they are also implicated in various neurological disorders such as depression, bipolar disorder, and schizophrenia. As a result, MMPs represent promising therapeutic targets for these conditions. Current strategies for inhibiting MMPs primarily focus on several approaches: chelating zinc ions to act as endogenous inhibitors, binding to allosteric sites to alter MMP conformation, simultaneously targeting both allosteric sites and metal chelation, and utilizing inhibitory antibodies to disrupt the interaction between MMPs and cell surface binding ligands (Chaves Filho et al., 2022) (Table 3). Minocycline is among the most extensively studied drugs in this context. It is a semi-synthetic tetracycline antibiotic known for its high lipophilicity (Yong et al., 2004), enabling it to cross the BBB. Minocycline not only possesses extensive antibacterial properties but also offers anti-inflammatory, neuroprotective, and MMP inhibitory effects. While it inhibits a wide range of MMPs, minocycline particularly targets MMP-9 and its upstream inducer, EMMPRIN (Liu et al., 2022). This action reduces the degradation of tight junction proteins, protects the BBB, and mitigates brain injury and neurological deficits following ICH (Wasserman and Schlichter, 2007).

**TABLE 3:** The inhibitors of MMPs.

Inhibitor name	Category	Mechanism of action and target	Characteristics and therapeutic potential
Minocycline	Tetracycline antibiotic	Inhibits MMP-9 and EMMPRIN, reduces degradation of tight junction proteins, protects BBB	Lipophilic, can cross the BBB, has anti-inflammatory and neuroprotective effects, reduces brain damage after hemorrhage
Doxycycline	Tetracycline antibiotic	Inhibits MMP-1, -2, -3, -7, -8, -13, broad-spectrum inhibition of MMPs activity	Broad-spectrum inhibitor, lacks specificity, may cause side effects
Hydroxamate inhibitors	Small molecule inhibitors	Chelates Zn^2+^ to inhibit the activity of multiple MMPs (e.g., BB-94/Batimastat, BB-1101, BB-2293, BB-2516/Marimastat)	Broad-spectrum inhibitors, but lack specificity, may cause side effects
GM6001, GM352, glyburide	Small molecule inhibitor	Inhibits the activity of multiple MMPs	Broad-spectrum inhibitor, lacks specificity, may cause side effects
JNJ0966	Highly selective inhibitor	Binds to the propeptide domain of pro-MMP-9, inhibits activation of MMP-9 without affecting other MMPs	Highly selective, does not affect other MMPs, potentially reduces side effects

Doxycycline, another derivative of tetracycline, functions as an inhibitor of various MMPs, specifically MMP-1, -2, -3, -9, and -13. Research conducted by Liu et al. and Yao et al. demonstrated that minocycline effectively downregulated the expression and activity of MMP-9 by targeting the PI3K/Akt signaling pathway. Notably, minocycline displayed a more pronounced inhibitory effect on MMP-9 mRNA and activity compared to MMP-2, while leaving TIMP expression unaffected. Furthermore, doxycycline has been shown to reduce the mRNA levels of MMP-2 and MMP-9, lower the protein concentrations of MMP-2, MMP-7, and MMP-9, and indirectly inhibit MMP activity through the upregulation of TIMP-1 expression (Yao et al., 2007; Lin et al., 2021). Hydroxamic acid-based inhibitors, including BB-94 (batimastat), BB-1101, BB-2293, and BB-2516 (marimastat), inhibit MMP activity by chelating Zn^2+^ (Wojtowicz-Praga et al., 1997), zinc chelation affects catalytic activity but not necessarily non-catalytic MMP functions. BB-94 and BB-2516 are both broad-spectrum MMP inhibitors. BB-94 inhibits MMP-1, -2, -3, -7, and -9 (Botos et al., 1996), while BB-2516 inhibits MMP-1, -2, -3, -7, -9, and -12 (Steward and Thomas, 2000). Additional compounds like GM6001 and GM352, as well as glibenclamide, have also demonstrated the capacity to inhibit MMPs. GM6001 primarily exerts specific inhibitory effects on MMP-9, MMP-2, and MMP-9 (Levine et al., 2014). CM352 is a short-half-life MMP inhibitor that, when given early, effectively prevents hematoma expansion within 24 h and continues to do so over a 14-day period in rats. Furthermore, it enhances both functional and neurological recovery (Rodríguez et al., 2017). Hypothermia has proven effective in attenuating BBB breakdown, reducing cerebral edema, preventing apoptosis, and alleviating neurological impairments. This therapeutic approach works by downregulating the expression of protease-activated receptor 1, MMP-9, and aquaporin-4 (AQP4) (Wang and Tsirka, 2005; Cui et al., 2012; Jiang et al., 2017). Most existing inhibitors are broad-spectrum, targeting the proteolytic activities of MMPs and other zinc-dependent proteases. This lack of specificity can lead to considerable side effects, rendering them unsuitable for therapeutic use. However, as our understanding of MMP structure evolves, the non-catalytic domains offer new avenues for the development of more refined inhibitors. A notable example is the work by Levin et al., who identified a highly selective compound JNJ0966. This compound binds to the pro-peptide domain of MMP-9, effectively preventing its activation while sparing other MMPs from inhibition (Levin et al., 2017). The ongoing investigation into EMMPRIN might lead to the creation of inhibitors specifically targeting it, such as antagonistic peptides-9 (AP9), berberine, resveratrol, and so on, which can consequently inhibiting MMPs (Liu Y. et al., 2021; Liu et al., 2022). The high specificity of monoclonal antibodies enables the creation of antibodies targeting different structural domains of MMPs. Additionally, protein engineering techniques can be utilized to design effective inhibitors of MMPs. Future research on MMP inhibitors will likely aim to enhance the specificity of binding sites and develop advanced smart drug delivery systems that are highly selective (Li K. et al., 2020). Therapeutic strategies for MMP inhibition require careful consideration of their dual roles. Suppressing MMP activity, particularly during the subacute and chronic phases, may inadvertently impede their critical functions in neurorepair and angiogenesis, potentially compromising long-term functional outcomes (Zhao et al., 2006). The extensive expression of MMPs and their diverse physiological functions throughout the body raise important concerns regarding potential off-target effects beyond the central nervous system. Many available inhibitors lack specificity, complicating efforts to accurately link observed intervention responses to distinct MMP activities and obstructing the establishment of clear causality. Importantly, while some therapies show promise in experimental models, their effectiveness may not carry over into clinical practice for patients suffering from intracerebral hemorrhage. At present, there are considerable gaps in our understanding of the safety and systemic tolerability linked to long-term or potent MMP inhibition, we can develop preclinical models (e.g., conditional knockouts or time-controlled inhibitors) as potential solutions (Vandenbroucke and Libert, 2014). Striking a balance between mitigating acute injury and maintaining reparative mechanisms presents a significant challenge for current research and is a critical priority for future development. This could involve creating highly selective inhibitors or carefully timing intervention windows to optimize therapeutic outcomes (Rempe et al., 2016).

## 5 Future directions and knowledge gaps

MMP-9 and MMP-3 exert significant detrimental effects during the acute phase, making them key targets for intervention (Alvarez-Sabín et al., 2004). MMP-2 exhibits mildly detrimental effects during the acute phase; however, it may transition to beneficial effects during the subacute phase. This indicates that its role is influenced by the timing of its activity (Castellazzi et al., 2010). Approximately 7 days may represent a critical transition point where MMPs shift from “destruction” in the acute phase to “repair” in the subacute phase. Future therapies should consider time-window selective inhibition. Current research primarily focuses on MMP-9 and MMP-2 (Xue and Yong, 2020), with the specific temporal roles of other MMP subtypes in secondary brain injury and post-hemorrhagic repair processes remaining unclear. Current pharmacological studies on MMP regulation primarily offer indirect evidence of their effects on target enzyme activity. To unlock the full potential of MMP-targeted therapies, it is crucial to specifically modulate individual MMP subtypes within defined spatiotemporal contexts. Additional research is necessary to clarify the interactions between MMPs and their surrounding molecular and cellular environments, identify optimal intervention timepoints, and create highly selective, low-toxicity inhibitors using innovative drug discovery platforms (Rempe et al., 2016). Only then can these therapeutic strategies be effectively integrated into routine clinical practice. Previous studies have largely concentrated on the detrimental effects of MMPs during the acute phase of ICH. Future investigations should aim to clarify the dynamic changes and regulatory mechanisms of MMPs throughout the acute, subacute, and chronic phases of hemorrhage, with particular attention to their interactions with processes such as BBB disruption and repair, neuroinflammation, and angiogenesis. Research on MMP inhibitors should prioritize the development of highly selective, subtype-specific agents. By combining this with precise timing of intervention and targeted delivery systems, we can transition MMP-targeted therapies from the laboratory into clinical practice.
